# Detection of quorum-sensing-related molecules in *Vibrio scophthalmi*

**DOI:** 10.1186/1471-2180-8-138

**Published:** 2008-08-13

**Authors:** Cristina García-Aljaro, Leo Eberl, Kathrin Riedel, Anicet R Blanch

**Affiliations:** 1 Department of Microbiology, University of Barcelona, Diagonal 645, E-08028 Barcelona, Spain; 2 Department of Microbiology, University of Zurich, Zollikerstrasse, 107, CH-8008-Zurich, Switzerland

## Abstract

**Background:**

Cell-to-cell communication (also referred to as quorum sensing) based on *N*-acyl-homoserine lactones (AHLs) is a widespread response to environmental change in Gram-negative bacteria. AHLs seem to be highly variable, both in terms of the acyl chain length and in the chemical structure of the radicals. Another quorum sensing pathway, the autoinducer-2-based system, is present both in Gram-positive and Gram-negative bacteria. In this study the presence of signal molecules belonging to both quorum sensing signalling pathways was analysed in the marine symbiotic species *Vibrio scophthalmi*.

**Results:**

Three AHL-like signal molecules were detected in *V. scophthalmi *supernatants with the *Agrobacterium tumefaciens *sensor assay. This observation was further supported by the decrease in the presence of these signal molecules after cloning and expression of lactonase AiiA from *Bacillus cereus *in the *V. scophthalmi *strains. One of the signal molecules was identified as *N*-(3-hydroxy dodecanoyl)-L-homoserine lactone. *V. scophthalmi *was also shown to carry a functional LuxS synthase. The coding sequence for a *luxS*-like gene was obtained showing a maximum similarity of 78% with *Vibrio vulnificus*. Analysis of the translated sequence revealed that the sequenced *luxS *gene carried the conserved domain, which is common to *luxS *sequences found in other species, and which is essential for LuxS enzymatic activity.

**Conclusion:**

The data are consistent with the presence of quorum-sensing signal molecules from both AHL- and autoinducer 2-based quorum sensing systems in *V. scophthalmi*, which are homologous to others previously described in various *Vibrio *species. How this bacterium interacts with other bacteria and eukaryotic cells to compete ecologically with other intestinal bacteria present in the fish *Scophthalmus maximus *warrants further investigation.

## Background

Quorum sensing is an extensive system whereby bacteria can communicate not only with members of their own species, but also with other species to coordinate their behaviour in response to population density, thereby modulating the expression of specific genes in a population-density dependent manner. This phenomenon, known as quorum sensing (the term proposed by [1], relies on the production and sensing of one or more secreted low-molecular-mass signal molecules, the extracellular concentration of which is related to the population density of the producing organism. Once the signal molecule has reached a critical concentration, equivalent to a certain cell density, the population elicits a particular response. This mechanism has been demonstrated in various Gram-negative and Gram-positive bacteria. In addition, various biological functions have been shown to be regulated by these molecules either directly or indirectly, including bioluminescence in *Vibrio *species [2], the expression of virulence factors (*Erwinia *sp., *Pseudomonas aeruginosa*, *Burkholderia cepacia*, *E. coli *O157:H7, *Vibrio cholerae*) [3-7], and biofilm formation and swarming motility in *P. aeruginosa *and *B. cepacia *[8,5], among others.

Two main types of quorum-sensing signalling pathways have been described for Gram-negative bacteria: the acylhomoserine lactone (AHL)-based system and the autoinducer 2-based system, which is described below. The first AHL-based quorum-sensing system described was involved in the control of bioluminescence in *V. fischeri *via the LuxR/I system [9,10]. LuxI synthesizes the diffusible signal molecule *N*-(3-oxohexanoyl)-L-homoserine lactone, which increases in concentration as the cell density increases. At a critical concentration, LuxR first binds the signal molecule and then activates the expression of target genes. Genes homologous with this system have been found in several Gram-negative bacteria, although *N*-acyl-homoserine lactones (AHLs) seem to be highly variable, both in terms of the acyl chain length and in the chemical structure of the radicals. Moreover, different AHLs synthases have been described in different quorum sensing signalling pathways. For instance, in *V. harveyi *a 3-hydroxy-C4-HSL is synthesized by the LuxM synthase and received by luxN protein. The genes coding for these proteins show no homology to the previously described LuxR/I quorum sensing system. The complex quorum sensing circuit also differ from other previously reported systems as stated below [11]. It has also to be noted that some bacteria have been shown to express enzymes that are able to interfere with AHL-based quorum-sensing systems from other species: acylases, which remove the side chain from the lactone ring from the AHLs by hydrolyzing the connecting amide bond between the ring and the side chain [12] and lactonases, which hydrolyze the lactone ring of the AHLs [13].

On the other hand, bacteria have been shown to possess a different mechanism for interspecies communication, namely the LuxS (AI-2) system previously described [14]. The LuxS is an autoinducer synthase produced by many diverse bacteria, including both Gram-negative and Gram-positive species [14]. *luxS *encodes an S-ribosyl homocysteinase, which converts S-ribosyl homocysteine from the methyl cycle into homocysteine (which is then returned to the active methyl cycle), as well as the autoinducer 2 [15], a borate diester of the cyclized 4,5-dihydroxy-2,3-pentanedione [16]. The AI-2 system has been shown to regulate several physiological activities including pathogenicity (though the control of toxins and other virulence expression factors), motility, biofilm formation, antibiotic production, and bioluminescence, among others (see review [15]).

*Vibrio scophthalmi *belongs to the highly heterogeneous *Vibrionaceae *family, which is comprised of 64 species, including both free-living and symbiotic species characterized by mutualistic or pathogenic relationships [17]. *V. scophthalmi *was first described in association with turbot (*Scophthalmus maximus*), being the most abundant *Vibrio *species found in intestinal bacterial populations [18]. It has been shown that *V. scophthalmi *maintains host specificity, despite showing a different phenotype pattern in 15–57 day-old larvae [19]. This fact, together with the finding that this organism is not the most abundant *Vibrio *spp in those waters surrounding the fish, led us to hypothesise that the benefits of turbot gut colonization by *V. scophthalmi *could be dependent upon a quorum-sensing system that responded when a sufficient number of bacteria were present in the gut.

Some symbiotic species belonging to the *Vibrionaceae *family have been shown to regulate bioluminescence through a complicated network of quorum-sensing systems involving the *lux *genes. Homologues to the LuxR/I system have been described for *V. fischeri*, *V. anguillarum*, *V. cholerae*, *V. logei *and *V. parahaemolyticus *[20-23]. In *V. harveyi*, the quorum-sensing as stated above, is a complex circuit consising of a multichannel two-component phosphorelay signal transduction pathway. In this bacterium the LuxR/I system is not present. Instead, the LuxM/N system works in parallel as well as the CqsA/S system with the LuxS/PQ to regulate a transcriptional activator, LuxR (not similar to other LuxR-type quorum-sensing proteins). [11]. Another complex quorum system was also described in *V. cholerae *[24].

Here we study the quorum-sensing signal synthases in *V. scophthalmi*, focusing on the two most common signal molecules in Gram-negative bacteria described above (AHLs and AI-2). The expression of AHLs was analyzed by thin layer chromatography (TLC) using different AHL sensor strains. Recombinant *V. scophthalmi *strains expressing an *aiiA *gene that coded for a lactonase were obtained and the loss of AHL production investigated. The presence of AI-2 synthase was analyzed by using the *V. harveyi *autoinducer bioluminescence bioassay [2] while the *luxS *gene, which is known as the synthase for AI-2, was detected by PCR and then sequenced.

## Results and discussion

### Identification of AHLs autoinducers

**Figure 1:**
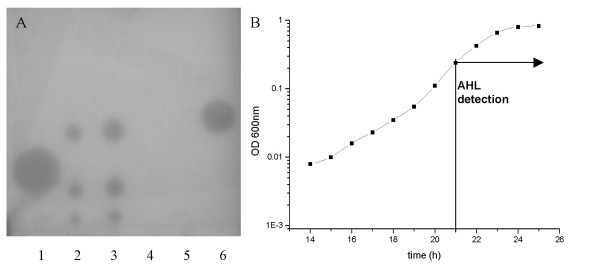
**Detection of AHLs by thin layer chromatography (TLC) with the *A. tumefaciens *NTL4 (pZLR4) strain used as a biosensor (A) and AHL detection along the growth curve in mTSB medium (B)**. A) lane 1: C8-HSL standard (Fluka); lane 2: *Vibrio scophthalmi *A089 strain; lane 3: *V. scophthalmi *A102 strain; lane 4: Negative control, mTSB alone; lane 5: Negative control, no sample added; 6: C6-HSL standard (Fluka). B) Arrow indicates the beginning of the detection of the three signalling spots, which were detected at the same time from the mid-log phase.

The *V. scophthalmi *strains A089 and A102 were grown overnight in different growth media (mLB, mTSB, mPW, and AB) and the supernatant was analysed for the presence of AHL-like molecules. Three different signal spots were detected in the TLC assay performed with the *A. tumefaciens *strain NTL4 (pZLR4) (Fig. 1a), which were not present in the medium alone used as negative control, suggesting the presence of three AHL-like molecules in the supernatant of *V. scophthami*. The retention factor (Rf) of the three AHL-like molecules was 0.62 (± 0.055), 0.46 (± 0.038) and 0.25 (± 0.018), from higher to lower Rf, respectively, as a result of three independent experiments. These three AHL-like molecules detected by the *A. tumefaciens *sensor strain were produced with any of the employed growth medium: mLB, mTSB, mPW, and AB (data not shown). Moreover, as shown on Fig. 1b, all three molecules were detected by TLC in the mid-exponential growth phase and maintained over the stationary phase.

In order to further characterise these molecules, different AHL standards and different sensor strains, which have been shown to detect previously described AHLs, were used. The autoinducer bioassay for the detection of AHLs, which employed the BB886 *V. harveyi *sensor strain, showed no induction of the luminescence signal compared with the negative control. Neither signal was detected with the *Chromobacterium violaceum *CV026 or the *Pseudomonas putida *F117 (pAS-C8) and *P. putida *F117 (pKR-C12) sensor strains. This may be related to the varying sensitivities of these sensor strains to a wide range of AHLs. The *C. violaceum *forward bioassay [25], detects preferably short chain AHLs, preferably HHL, although it has been also shown to detect 3 hydroxy-C6-HSL [26] but not longer chain AHLs. The *P. putida *F117 (pAS-C8) sensor strain is sensitive to C8-HSL and *P. putida *F117 (pKR-C12) is highly sensitive to C12-HSL, 3-oxo-C12-HSL, C10-HSL, and 3 oxo-C10-HSL [27]. The fact that it was not possible to detect any signal in the *V. harveyi *BB886 autoinducer bioassay suggests that the putative AHLs from *V. scophthalmi *detected with the *A. tumefaciens *assay are sufficiently different from that of *V. harveyi *to be recognised.

### Chemical characterisation of the AHL-like molecules

**Figure 2:**
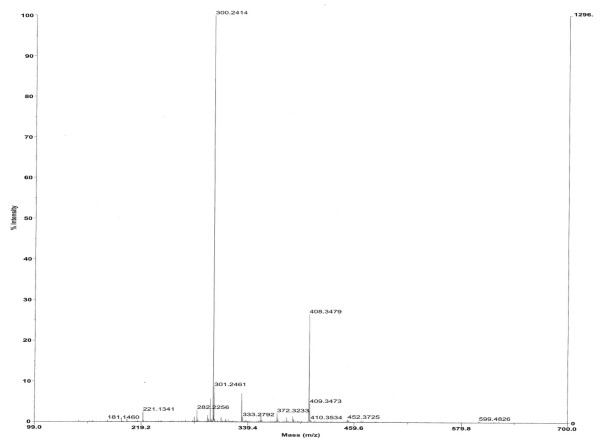
**Chromatograph obtained from the lower AHL signalling spot from the TLC**. A peak of 300.2414 was obtained with a Mariner TOF MS, whose exact mass was later confirmed as m/z 300.2167 after HPLC and mass spectrometry with electrospray ionisation analysis. This molecule matched the empirical formula C16H29NO4 (with an error of 0.39 ppm) for M and hypothetically corresponds to a molecular structure of a hydroxylated-C12HSL. A peak of 282.2256 was also observed which could represent loss of OH group due to fragmentation of the molecule, and a 599.4826 peak, an aggregation of two molecules. Other peaks were originated from components of the medium used for growing the bacterium.

Detailed analyses of the AHL-like molecules detected were carried out exclusively with the strain *V. scophthalmi *A102, since both strains presented the same TLC pattern. Two different culture media, mTSB and mPW, were evaluated for growing *V. scophthalmi *A102 in order to carry out the extraction of AHLs. Due to the high background observed with the mTSB medium, we decided to use the mPW culture medium. Only that extract corresponding to the lower Rf spot resulted in a structure composition similar to an AHL, which hypothetically corresponded to an *N*-(3-hydroxy dodecanoyl)-L-homoserine lactone (N-3-hydroxy-C12-HSL). This molecule matched the empirical formula C_16_H_29_NO_4 _+ H^+ ^with mass (m/z) 300.2167 and an error of 0.39 ppm (see Fig. 2). As far as we know, this constitutes the first description of an AHL in *V. scophthalmi*; moreover, it has proven different from those produced by other *Vibrio *species. It was not possible to identify any recognizable AHL-like structures in the other signalling spot extracts, even after carrying out four independent extraction experiments.

The high background levels caused by other molecules in relation to the amount of AHL produced of the other two putative AHLs may have hindered the detection of these molecules. However it can not be discarded that these two signalling spots that were detected with the *A. tumefaciens *sensor strain, but not detected by HPLC coupled with mass spectrometry, might indeed not represent AHLs but AHL-derived or AHL-regulated molecules, that would induce non-specifically the *A. tumefaciens *sensor strain, since this sensor is known to be activated non-specifically.

Acylhomoserine lactone signal synthases have been studied in other *Vibrio *species. This species have been shown to produce several AHLs, which are produced by different synthases: *V. anguillarum *(hexanoyl homoserine lactone and 3-hydroxy-hexanoyl homoserine lactone synthesized by VanM (homologous to the LuxM from *V. harveyi*) and the 3-oxo-decanoyl homoserine lactone, synthesized by VanI, a LuxI homologue) [26], *V. fischeri *(3-hydroxy-butanoyl homoserine lactone, synthesized by LuxI, and octanoyl homoserine lactone synthesized by AinS, a LuxM homologue) [28], *V. harveyi *(3-hydroxy-butanoyl homoserine lactone, synthesized by LuxM) [11], *V. vulnificus *(butanoyl homoserine lactone, 3-oxo-decanoyl homoserine lactone, 3-oxo-dodecanoyl homoserine lactone, and in minor amounts, hexanoyl homoserine lactone, 3-oxo-octanoyl homoserine lactone, and 3-oxo-tetradecanoyl homoserine lactone, synthesized by a LuxI homologue), and *V. salmonicida *(3-oxo-hexanoyl homoserine lactone and hexanoyl homoserine lactone, synthesized by a LuxI homologue) [29].

### Heterologous expression of *aiiA *in *V. scophthalmi*

**Figure 3:**
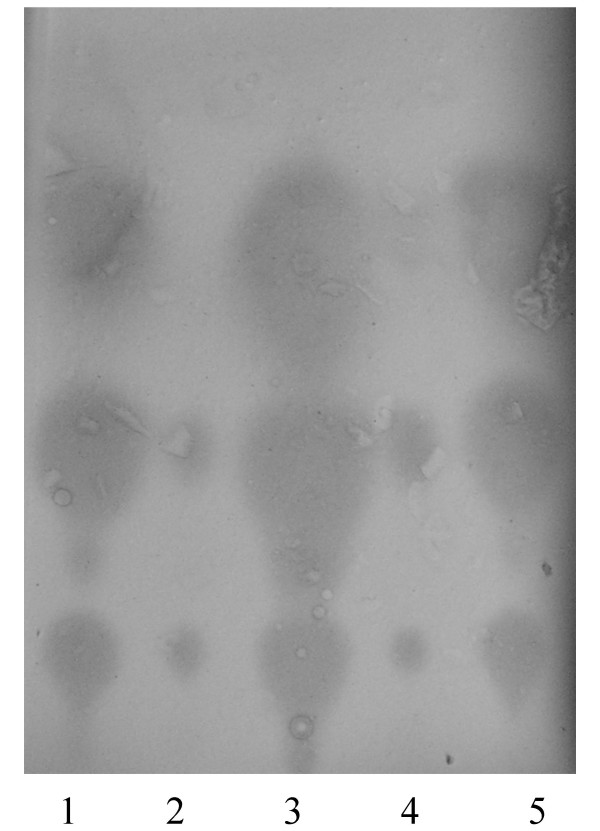
**Effects of *aiiA *gene introduction in *V. scophthalmi *in the reduction of the AHL levels detected in a TLC assay following exposure to the sensor strain *A. tumefaciens *NTL4(pZLR4)**. Lane 1: *V. scophthalmi *A089; lane 2: *V. scophthalmi *A089 pACYC184-*aiiA*; lane 3 *V. scophthalmi *A102; lane 4: *V. scophthalmi *A102-*aiiA*; lane 5: *V. scophthalmi *A102 control strain with the pACYC184 plasmid, but without the *aiiA *gene.

In order to confirm the presence of AHLs, AHL-derived or AHL-regulated substances in the supernatant of *Vibrio scophthalmi*, the lactonase gene *aiiA *from the *Bacillus cereus *strain A24 was subcloned into the pACYC184 plasmid and then electroporated into *E. coli *DH5α. This plasmid was subsequently transferred to the *Vibrio *strain by conjugation. We utilized two strains for those experiments addressing heterologous expression: *V. scophthalmi *A089 and *V. scophthalmi *A102. As measured by both, diffusion assays in agar plates and TLC, expression of the *aiiA *gene in both strains reduced autoinducer accumulation (Fig. 3). We constructed a control strain by conjugating the pACYC184 plasmid without the *aiiA *gene. As this control strain showed no AHL reduction, we attributed the decreased AHL production in recombinant strains to heterologous expression of the lactonase gene, confirming that these three putative autoinducers possessed an AHL-like structure or were AHL-regulated.

This finding indicates not only that lactonase is expressed in the *Vibrio *strains, but also that its activity results from the hydrolysis of AHL lactone bonds, as has been reported in other bacterial species [30]. We demonstrate that this lactonase is able to degrade hydroxylated AHLs, such as N-3-hydroxy-C12-HSL, thereby increasing its substrate spectrum. These findings are consistent with other studies that have shown that lactonases from the *Bacillus *genus are characterized by certain variability in their capacity to degrade different AHLs [13]. This is true for the lactonase from the *B. cereus *strain, which reduced the concentrations of C6-HSL, oxo-C12-HSL and C4-HSL from *P. aeruginosa *to non-detectable levels [4].

### Detection of a LuxS synthase in *Vibrio scophthalmi*

**Figure 4:**
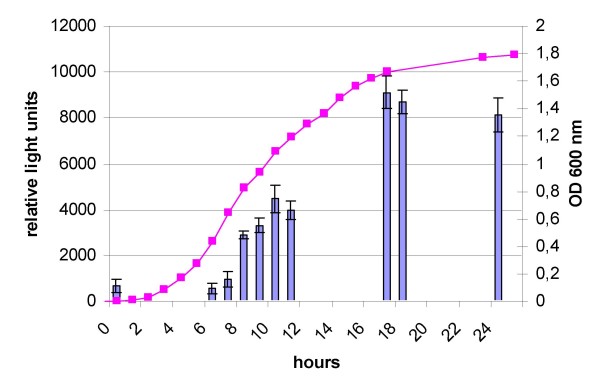
**Detection of LuxS activity in the supernatant of the *V. scophthalmi *A089 strain by a bioluminescence assay using the *V. harveyi *reporter strain BB170**. Bars, relative light units produced by stimulation of the *V. harveyi *BB170 reporter strain after addition of supernatant from *V. scophthalmi *A089, taken at different time points; squares, growth of *V. scophthalmi *as measured by OD at 600 nm.

The presence of LuxS activity in the supernatants from the *V. scophthalmi *A089 strain was assessed by using the *V. harveyi *reporter strain BB170 in a bioluminescence assay. Two negative controls were included, consisting of the incubation medium alone, and the medium with the sensor strain. Although we tested two different culture media, mTSB and the mPW, the former was discarded due to the fact that some of the components of the mTSB induced the sensor strain producing a false positive reaction. As shown in Fig. 4, the maximum induction level for the reporter strain was obtained in those supernatants from *V. scophthalmi *A089 grown in mPW medium, which corresponded to the late logarithmic phase. Here, we recorded a maximum induction value of 35% with respect to the positive control used in this assay, *V. harveyi *BB120.

**Figure 5:**
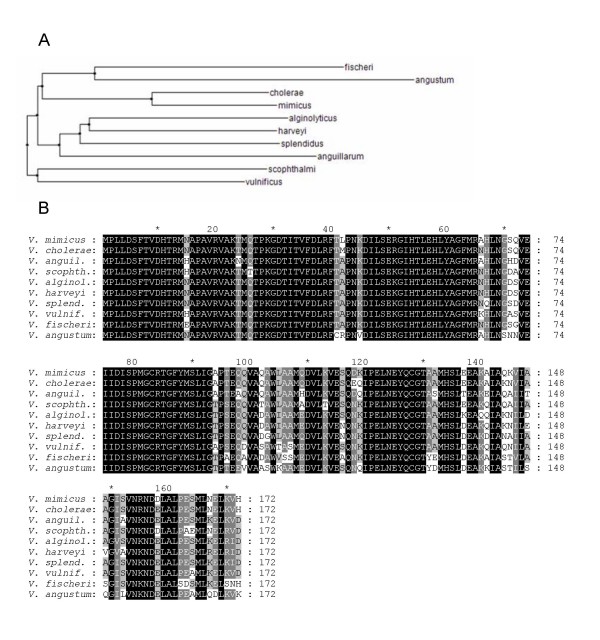
**Phylogenetic tree based on *luxS *homologous sequences from different *Vibrio *species**. A) Phylogenetic tree based on the *luxS *gene sequence following the neighborjoining clustering method using ArboDraw. B) Protein coding sequence alignment of different homologous LuxS protein sequences. Alignment of the translated genes was carried out using the Multalin program [41] and edited with Gendoc. Accession numbers:*V. mimicus*, [GenBank:AB232376], *V. cholerae *[GenBank:AB114425], *V. anguillarum *[GenBank:DQ466077], *V. algynolyticus *[GenBank:AY391122], *V. harveyi *[GenBank:AF120098], *V. splendidus *[GenBank:NZ_AAMR01000072], *V. vulnificus *[GenBank:AF305637], *V. fischeri *[GenBank:CP000020], *V. angustum *[GenBank:NZ_AAOJ01000005].

These molecules were likely to be synthesized by the AI-2 synthase LuxS, whose coding sequence was determined for *V. scophthalmi*, and which exhibited a maximum similarity to the *V. vulnificus luxS *gene (78%). The coding sequence consisted of 519 bp, which is common to other *luxS *sequences from various *Vibrio *species. This sequence was submitted to the GenBank database under the accession number [GenBank:EF363481]. A phylogenetic tree of different *Vibrio luxS *sequences is shown on Fig. 5a. The translated sequence revealed that the sequenced *luxS *gene carried a conserved domain common to *luxS *sequences found in other species, which is essential for LuxS enzymatic activity (Fig. 5b, sites 54–58, HTLEH). The sites 118–126, NKIPELNEY, representing a hypothetical phosphorylation site [31], was also conserved. This domain has already been detected in *luxS *homologous sequences from *Escherichia coli*, *Salmonella typhimurium*, *Yersinia *sp., and *Shigella sonnei*, among others [31]. All of these findings support the contention that *V. scophthalmi *possesses a functional LuxS synthase.

## Conclusion

This study confirms the presence of quorum-sensing signal molecules in *V. scophthalmi *from the AHL-based and type 2 (AI-2)-based systems. How this bacterium interacts with other bacteria and eukaryotic cells to compete ecologically with other intestinal bacteria present in the fish *Scophthalmus maximus *warrants further investigation. In this way, *in vivo *and *in vitro *analyses will be needed, so that we can understand precisely how these different networks are connected.

## Methods

### Bacterial strains, plasmids, and culture conditions

The bacterial strains and plasmids used in this study are listed in Table 1. All strains were grown at 30°C, agitated at 180 rpm in LB for *Escherichia coli*, or modified (*i.e.*, the same medium with a final concentration of 2% NaCl) TSB (mTSB) (Difco, Barcelona, Spain) or LB (mLB) [32] for *Vibrio *strains, unless otherwise stated. In order to detect AHLs, different media were used for growing *V. scophthalmi*, mTSB, mLB, AB (autoinducer bioassay medium [2], and modified peptoned water (mPW) (*i.e.*, peptone water containing 2% NaCl (w/v)). When necessary, the concentration of the antibiotics used was as follows: 25 μg/ml tetracycline, 10 μg/ml chloramphenicol and 25 μg/ml chloramphenicol for counter selecting *Vibrio *recombinants and *E. coli*, respectively, and 100 μg/ml ampicillin for *E. coli*. TCBS medium (Oxoid S.A., Madrid, Spain) was used for selection of specific *Vibrio *strains.

**Table 1:** Bacterial strains and plasmids used in this study

**Strains or Plasmids**	**Characteristics**	**Reference or source**
**Strains**		
*Vibrio scophthalmi *A089	Turbot isolate, CECT 4638T	[18]
*Vibrio scophthalmi *A102	Turbot isolate	[18]
*Vibrio scophthalmi *A089-aiiA strain	A089 strain with the *aiiA *gene cloned into pACYC184 plasmid, Cl^r^	This study
*Vibrio scophthalmi *A102-aiiA strain	A102 strain with the *aiiA *gene cloned into pACYC184 plasmid, Cl^r^	This study
*E. coli *DH5α	F^-^Φ80d lacZΔM15endA1hsdR17(r_k_^- ^m_k_^-^) *supE44 thi-1 gyrA96*Δ*(lacZYA-argF)*	[37]
*E. coli *HB101 (pRK2073)	Mobilizing strain; Sp^r^	Biomedal
*E. coli *One Shot cells	Used for transformation Experiments	Invitrogen
**Sensor strains**		
*A. tumefaciens *NTL4(pZLR4)	AHLs sensor strain, Gm^r^	[34]
*C. violaceum *CV026	AHLs sensor strain	[25]
*V. harveyi *BB866	AI-1 indicator strain, BB120 *luxP*::Tn*5*	[2]
*V. harveyi *BB170	AI-2 indicator strain, BB120 *luxN*::Tn*5*	[42]
*V. harveyi *BB120	wild-type strain	[2]
*P. putida *F117 (pAS-C8)	AHLs sensor strain, Gm^r^	[27]
*P. putida *F117 (pKR-C12)	AHLs sensor strain, Gm^r^	[27]
**Plasmids**		
pCR2.1-TOPO	TA cloning vector; Amp^r^, Km^r^	Invitrogen
pGEM-T-easy	TA cloning vector; Amp^r^	Promega
pACYC184	Broad-host range expression vector, Tc^r^, Cl^r^	[36]
pACYC184-aiiA	Broad-host range expression vector carrying the *aiiA *gene from *Bacillus cereus *strain A24; Cl^r^	This study
pME6863	Broad-host range expression vector carrying the *aiiA *gene from *Bacillus cereus *strain A24; Tc^r^	[4]

Antibiotic abbreviations used in this study: Ampicillin (Amp), Kanamycin (Km), Spectinomycin (Sp), Chloramfenicol (Cm), Trimethoprim (Tri), Tetracycline (Tc); Spanish Type Culture Collection (CECT).

### Detection of AHLs by thin layer chromatography (TLC)

AHLs were extracted as follows: for analytical TLC, 5 ml free-cell overnight cultures of the relevant strains were mixed 1:1 with acidified ethyl acetate. The solvent phase was recovered with a pipette and residual water in the extract was eliminated by adding anhydrous magnesium sulphate. The extract was then filtered through a Whatman paper 3 MM, dried with nitrogen flux air, and resuspended in 20 μl of ethyl acetate. For preparative TLC, we used the same procedure except that the extract was rotary evaporated instead of nitrogen air-dried.

The presence of AHLs in the extracts was tested by C_18 _reverse-phase TLC (Uniplate RPS Reverse-Phase Hydrocarbon Impregnated Silica Gel; Analtech, Sigma-Aldrich, Barcelona, Spain) using methanol/water (60:40, v/v) [33]. Different QS-sensor strains were employed to test for the presence of AHLs (Table 1). Briefly, the plates were dried and overlaid with 50 ml of soft agar; LB for *C. violaceum*, and AB for *A. tumefaciens*, containing 20 ml of an overnight culture of the corresponding sensor strain to give a final concentration of 0.7% agar (w/v). For *A. tumefaciens*, gentamicin (30 μg/ml) and X-Gal (80 μg/ml) were added. The plates were incubated for 24 h at 30°C; the emergence of blue spots was considered a positive signal for the presence of AHLs [34]. For *Pseudomonas putida *sensor strains (Table 1) LB with 25 μg/ml gentamicin was used instead. The presence of AHLs was determined by emission of fluorescence that was detected by illumination with blue light by using an HQ 480/40 filter (AHF-Analysentechnik) in combination with an halogen lamp as a light source in a dark box equipped with a light sensitive camera (Hamamatsu photonics, Herrsching, Germany) with a Pentax CCTV camera lens and an HQ 535/20 filter.

### Identification of AHL molecules

Extracts were prepared from 1 l of overnight-free-cell culture supernatant extracted with ethyl-acetate, as described above. It was then concentrated to 1 ml by rota-evaporation. TLC was subsequently carried out and the signalling spots were analyzed separately by cutting off the silica from the plates and extracting the AHLs in 1 ml of ethyl-acetate. The silica was then discarded and the solvent phase was resuspended in 0.5 ml of methanol prior to analysis.

Identification of AHLs was carried out at the "*Serveis Cientifico-Tècnics*" of the University of Barcelona by HPLC coupled with Mass Spectrometry and Electrospray Ionisation using an API™ – Mariner TOF device (Applied Biosystems) based on the protocol described previously [35].

### Empirical formula confirmation

Confirmation of the AHL formula was performed at the "*Unitat d'Espectrometria de Masses*" of the University of Barcelona. The system used consisted of an Agilent LC/MSD TOF coupled to an Agilent 1100 Series HPLC. The TOF was equipped with a dual-nebulizer electrospray source, which allows continuous introduction of an internal reference mass compound at a low concentration for automatic internal mass calibration. The instrument scanned from 100 to 1000 m/z. This range included two reference mass compounds: Purine m/z: 121.050873 and HP-0921 m/z: 922.009798. Resolution 10000 at m/z: 922.009798. Instrumental parameters were as follows: Positive ESI Capillary 4 KV; Fragmentor 215 V; Gas temperature: 300°C, Nebulizer gas (N_2_) 15 p.s.i. (1 p.s.i. = 6.9 kPa), drying gas (N_2_) 7 l min^-1^.1 μl of the sample, extracted as indicated above, was pumped into the source at a flow rate of 0.2 ml/min with a mixture of H_2_O:CH_3_CN 1:1 1% (v/v) formic acid as eluent. Spectra from the appropriate chromatogram were averaged and the suspected molecular ion was then selected (m/z: 300.2170). Possible formulas were determined by using the elemental composition calculator built into the data analysis software, and applying constraints on the elemental composition.

### DNA isolation and nucleotide sequencing

Plasmid DNA was isolated with the Qia Prep Spin Miniprep Kit (Qiagen Inc., Valencia, California, United States). Genomic DNA was isolated with the Qiagen DNA Blood Kit (Qiagen). DNA sequencing was performed with the Big Dye Terminator Cycle Sequencing Ready Reaction Kit 3.1 (Applied Biosystems).

### Cloning of the *aiiA *gene on the broad host-cloning vector pACYC184

The *aiiA *gene from the *Bacillus *cereus A24 strain, coding for a lactonase, was subcloned from the pME6860 plasmid onto the pACYC184 plasmid [36], following a variation of the method previously described [4]. Briefly, the *aiiA *gene was amplified by PCR (3 min at 95°C; 21 cycles of 1 min at 95°C, 1 min at 50°C, 2 min at 72°C; 10 min at 72°C) from the DNA plasmid pME6860. The primers used for this amplification were designed as follows: aiiA-F-Bam: ACgTggATCCCgC**AggA**TCCATATgACAgTAAAgAAgCTT and aiiA-R-Sal: gCTggTCgACCgTCgACTATATATATTCAgggAA. Restriction sites for *Bam*HI and *Sal*I are underlined and the ribosome-binding site is written in bold. The PCR product was subcloned into the pGEM-T-easy vector (Promega, Barcelona, Spain) following the manufacturer's instructions. *E. coli *strains carrying the vector construction were screened on LB plates containing 100 μg ampicillin ml^-1 ^(Sigma Aldrich) and 50 μg 5-bromo-4-chloro-3-indolyl-β-D-galactosidase (X-Gal)/ml (Invitrogen, Barcelona, Spain) used for the blue-white selection. The resulting plasmid was electroporated into *E. coli *DH5α. The pACYC184-*aiiA *vector was constructed by cloning the *aiiA *gene from the pGEM-T-easy vector by sequentially cleaving with the *Bam*HI and *Sal*I restriction enzymes and ligating into the pACYC184 vector [37]. The *aiiA *gene was then sequenced in both strands to ensure that no mutations had occurred during the PCR reaction. The construct pACYC184-aiiA expressed *aiiA *from the vectors' constitutive tetracycline promoter.

### Conjugative plasmid transfer

Plasmids were delivered to *Vibrio *strains by triparental mating as previously described [38], using the helper strain *E. coli *HB101 (pRK2073), the donor strain *E. coli *(pACYC184-aiiA), and the *Vibrio scophthalmi *recipient strain A089 and A102. TCBS medium supplemented with 10 μg/ml chloramphenicol was used for counter selection of donor, helper, and untransformed recipient cells. The plates were incubated overnight and yellow colonies were selected and tested for the presence of the plasmid and the *aiiA *gene using the primers and conditions described above. Two recombinant strains were selected for further study: *V. scophthalmi *A089-aiiA and *V. scophthalmi *A102-aiiA. To confirm that the transconjugants were *V. scophthalmi*, sequencing of the 16S rRNA gene was also performed using the universal primers previously described [39].

### Effects of *aiiA *gene expression on recombinant strains

The effects of lactonase *aiiA *expression on the AHL accumulation levels in recombinant strains was analyzed visually by inducing the *A. tumefaciens *sensor strain by AHL-molecules separated by TLC, as described above. Growth kinetic analysis were also performed with the *V. scophthalmi *A089 and A102 strains, as well as the respective recombinants carrying the pACYC184-aiiA plasmid and the pACYC184 plasmid alone

### Bioluminescence assay for detecting AHL (AI-1) and autoinducer 2 (AI-2) quorum-sensing signal molecules

An overnight culture of *V. scophthalmi *was prepared and inoculated into fresh medium (1:100) in a final volume of 200 ml and incubated with agitation at 30°C, taking a 5-ml aliquot every hour in order to analyze the kinetics of autoinducer production. The absorbance of the culture was measured at 600 nm, centrifuged, and the supernatant was filtered through a 0.22 μm filter (Millipore Corporation, Barcelona, Spain) and kept at -20°C until analysis. *V. harveyi *indicator strains BB886 (sensor AI-1; sensor AI-2 defective) and BB170 (sensor AI-1 defective, sensor AI-2), as well as the positive control BB120 (AI-1 producer; AI-2 producer) [2], were used to detect the presence of autoinducers in the culture supernatants. Induction of the sensor strain was measured using a Lambda Fluoro 320 Plus reader (Bio-Tek Instruments, Winooski, Vermont, USA) and evaluated with the KC4 software (Bio-Tek, Instruments, Winooski, Vermont, USA). The results are expressed as the fold induction of the reporter strain over background when buffer or medium alone was added to the reporter. All assays were repeated at least three times.

### LuxS detection and sequencing of the gene

The presence of a LuxS-like molecule in the *V. scophthalmi *A089 strain was assessed by detection of LuxS activity following the bioluminescence method previously described [40] using the media and culture conditions stated in the previous section. Detection of the *luxS *gene was also performed by PCR. To this end, a set of degenerate primers was designed based on the sequence of previously reported *luxS *gene sequences recorded in the GenBank database with the following accession numbers: *V. vulnificus *[GenBank:NC_004459], *V. parahaemolyticus *[GenBank:BA000031], *V. alginolyticus *[GenBank:AY391122] *V. harveyi *[GenBank: GenBank:AF120098], and *V. fischeri *[GenBank:NC_006840]. The sequences of these primers within the luxS gene were as follows: LuxSF1-5'ATGCCWTTRYTVGAYAG 3' and LuxSR2-5' TCRTCRTTYTTRTTHAC 3'. The PCR conditions were as follows: 5 min at 95°C; 35 cycles: 1 min at 95°C, 1 min at 45°C, 1 min at 72°C; 10 min at 72°C. Two amplimers were obtained, and that which presented a similar size to the control strain was sequenced as described above. The similarity to the previously reported *luxS *sequences was then analyzed. In order to obtain the complete coding sequence of the gene, we designed an inverted PCR. For this purpose, genomic DNA was digested overnight with an *Nst*I restriction enzyme (Promega) and ligated with T4-DNA ligase (Invitrogen). The resulting ligated DNA molecules were amplified using a new set of primers: the LuxSF3 5' GAGCTGAATGAATACCA 3', designed from the previously sequenced amplimer, and a newly degenerated primer based on the sequences described above, LuxSR1 5' CCCATHGGYGADATATC 3'. The same PCR conditions were used except that the annealing temperature was set at 50°C. The resulting amplimer was sequenced using the same primers described above (LuxSF3 and LuxSR1), producing an overlapping sequence to the amplimer previously obtained, which allowed us to sequence the entire gene (519 bp). The complete gene sequence was confirmed by PCR using the primer previously described LuxSF1 and primer LuxSRseq, 5'-TTAATCGACGCGAAGCTCAT 3', which was located downstream from the *luxS *gene coding sequence deduced from the obtained sequence.

## Authors' contributions

CG–A performed most of the experiments, analysed the data and wrote the manuscript. LE designed the luxS experiments, and helped with writing the manuscript. KR helped in the analysis of the data and writing the manuscript. ARB participated in the design and coordination of the study and helped in writing the manuscript. All authors have read and approved the final version of the manuscript.
